# The Silva Pattern-based Classification for HPV-associated Invasive Endocervical Adenocarcinoma and the Distinction Between In Situ and Invasive Adenocarcinoma: Relevant Issues and Recommendations From the International Society of Gynecological Pathologists

**DOI:** 10.1097/PGP.0000000000000735

**Published:** 2021-02-09

**Authors:** Isabel Alvarado-Cabrero, Carlos Parra-Herran, Simona Stolnicu, Andres Roma, Esther Oliva, Anais Malpica

**Affiliations:** Hospital de Oncología, CMN, SXXI, Instituto Mexicano del Seguro Social, Mexico City, Mexico (I.A.C.); Brigham and Women’s Hospital and Harvard Medical School (C.P.H.); Massachusetts General Hospital and Harvard Medical School (E.O.), Boston, Massachusetts; University of Medicine, Pharmacy, Sciences and Technology of Targu Mures, Targu Mures, Romania (S.S.); University of California San Diego, San Diego, California (A.R.); The University of Texas MD Anderson Cancer Center, Houston, Texas (A.M.)

**Keywords:** Endocervical, Adenocarcinoma, In situ, Invasive, Silva, Pattern, Staging, Lymph node, Metastasis, Treatment

## Abstract

The Silva pattern-based classification for human papilloma virus–associated invasive adenocarcinoma has emerged as a reliable system to predict risk of lymph node metastasis and recurrences. Although not a part of any staging system yet, it has been incorporated in synoptic reports as established by the College of American Pathologists (CAP) and the International Collaboration on Cancer Reporting (ICCR). Moreover, the current National Comprehensive Cancer Network (NCCN) guidelines include this classification as an “emergent concept.” In order to facilitate the understating and application of this new classification by all pathologists, the ISGyP Endocervical Adenocarcinoma Project Working Group presents herein all the current evidence on the Silva classification and aims to provide recommendations for its implementation in practice, including interpretation, reporting, and application to biopsy and resection specimens. In addition, this article addresses the distinction of human papilloma virus–associated adenocarcinoma in situ and gastric type adenocarcinoma in situ from their invasive counterparts.

Invasive adenocarcinoma accounts for 7% to 29% of cervical carcinomas 1–4 and its incidence appears to be rising in some countries 5–8. In this article, we discuss 2 relevant issues regarding this type of tumor: (1) the Silva pattern-based classification for human papilloma virus (HPV)-associated adenocarcinoma, as morphologically defined by the International Endocervical Adenocarcinoma Criteria and Classification (IECC) 9, and (2) its distinction from in situ adenocarcinoma. This work is based on a review of the literature and a collaborative effort by a subgroup of the Endocervical Adenocarcinoma Working Group of the International Society of Gynecological Pathologists (ISGyP). The levels of evidence and recommendations presented herein follow the 2009 and 2011 Oxford Centre for Evidence-Based Medicine (OCEBM) recommendations. Levels of evidence and grades of recommendation under “Prognosis” or “Diagnosis” categories were used, when applicable 10 (Table 1).

**TABLE 1:** Levels of evidence in scientific studies, from the centre for Evidence-based medicine, University of Oxford 10

Diagnosis
1a: Systematic review (with homogeneity) of Level 1 diagnostic studies; or a clinical decision rule with 1b studies from different clinical centers
1b: Validating cohort study with good reference standards; or clinical decision rule tested within one clinical center
1c: Absolute SpPins And SnNouts (An Absolute SpPin is a diagnostic finding whose Specificity is so high that a Positive result rules-in the diagnosis. An Absolute SnNout is a diagnostic finding whose Sensitivity is so high that a Negative result rules-out the diagnosis)
2a: Systematic review (with homogeneity) of Level >2 diagnostic studies
2b: Exploratory cohort study with good reference standards; clinical decision rule after derivation, or validated only on split-sample or databases
3a: Systematic review (with homogeneity) of 3b and better studies
3b: Nonconsecutive study; or without consistently applied reference standards
4: Case-control study, poor or non-independent reference standard
5: Expert opinion without explicit critical appraisal, or based on physiology, bench research or “first principles”
Prognosis
1a: Systematic review (with homogeneity) of inception cohort studies; or a clinical decision rule validated in different populations
1b: Individual inception cohort study with >80% follow-up; or a clinical decision rule validated on a single population
1c: All or none case-series
2a: Systematic review (with homogeneity) of either retrospective cohort studies or untreated control groups in randomized controlled trials
2b: Retrospective cohort study or follow-up of untreated control patients in a randomized controlled trial; or derivation of a clinical decision rule or validated on split-sample only
2c: “Outcomes” research
4: Case-series (and poor-quality prognostic cohort studies)
5: Expert opinion without explicit critical appraisal, or based on physiology, bench research or “first principles”
Grades of recommendation
A: Consistent level 1 studies
B: Consistent level 2 or 3 studies or extrapolations from level 1 studies
C: Level 4 studies or extrapolations from level 2 or 3 studies
D: Level 5 evidence or troublingly inconsistent or inconclusive studies of any level

## THE SILVA PATTERN-BASED CLASSIFICATION FOR HPV-ASSOCIATED ADENOCARCINOMA

### Introduction

Cervical cancer is staged according to the Fédération Internationale de Gynécologie et d´Óbstétrique (FIGO) system 11,12 using a combination of clinical, imaging, and pathology findings. The experience with this staging system, however, is based primarily on studies of squamous cell carcinoma, which is by far more common, and has been extrapolated to adenocarcinoma. As a result, both adenocarcinomas and squamous carcinomas are staged and treated similarly, although there is increasing evidence to suggest that adenocarcinomas show different epidemiology, prognostic factors, patterns of spread and failure after treatment compared with squamous cell carcinomas 13,14.

Staging of FIGO IA1, IA2, and IB1 invasive endocervical adenocarcinomas (EACs) is currently based on the depth of invasion 11,12. However, an accurate assessment of this parameter can be challenging in a variety of scenarios such as: (1) well-differentiated invasive adenocarcinomas without architectural complexity and no stromal reaction that are difficult to distinguish from in-situ adenocarcinoma, (2) tumors where it is not possible to separate the invasive from the in situ component, (3) polypoid lesions, and (4) specimens lacking proper orientation or integrity of the mucosal surface. In spite of these potential challenges, depth of invasion is a major determinant of treatment. According to the current National Comprehensive Cancer Network (NCCN) guidelines, patients with FIGO stage IA1 tumors that lack lymphovascular invasion (LVI) could undergo conservative treatment with conization and follow-up (if margins are negative) or simple hysterectomy when preservation of fertility is not required. Patients with FIGO stage IA2 tumors and those with IA1 tumors associated with LVI or with positive margins undergo radical surgery (radical hysterectomy, or alternatively large conization or radical trachelectomy as fertility preservation approaches); sentinel lymph node (SLN) mapping and/or pelvic lymph node (LN) dissection are also considered in this group of patients 15. Patients that undergo simple/radical hysterectomy or radical trachelectomy may experience surgical complications such as bladder dysfunction, vascular or ureteral injuries, and blood loss among others 16. In addition, 10% to 41% of patients treated with LN dissection can experience lower extremity lymphedema as postoperative morbidity 17,18. Importantly, the literature indicates that few patients with early FIGO stage tumors have evidence of LN metastasis, seen in <1% of patients with stage IA1 tumors and in ~2% of patients with stage IA2 tumors 19.

In an attempt to improve the current risk stratification system for patients affected by HPV-associated invasive cervical adenocarcinoma, a group of pathologists led by Dr Elvio Silva have proposed the use of a system based on the following histologic features: tumor-stromal interface, presence or absence of LVI, architecture and grade of cytologic atypia 20,21.

### The Silva Pattern-based Classification: Definitions

The Silva classification stratifies HPV-associated invasive endocervical adenocarcinoma into 3 patterns (A, B, C) based on the presence or absence of destructive stromal invasion, the degree of destructive stromal invasion (if present), the presence or absence of LVI, and grade of cytologic atypia. This classification system does not take into account the depth of invasion or the relationship of the tumor to large vessels in the cervical stroma (Table 2). The definitions and specific cut-offs presented herein have been established by consensus of the original group that defined the Silva classification 20,21.

**TABLE 2:** Silva pattern-based classification for HPV-associated invasive adenocarcinoma*

Pattern A
No destructive stromal invasion
Well-demarcated glands with rounded contours, commonly forming groups
No single cells or cell detachment
Complex intraglandular growth allowed (i.e. cribriform, papillae), < a 4× field (5 mm in diameter)
No solid growth or high-grade cytology
No lymphovascular invasion (LVI)
Irrelevant relationship to large cervical vessels or depth of the tumor
Pattern B
Localized (limited, early) destructive stromal invasion arising from well-demarcated glands (pattern A-like glands)
Individual, ragged glands or small clusters of tumor cells, separated from the rounded glands, usually in an inflamed or desmoplastic stroma
Foci may be single, multiple, or linear at the base of the tumor, < a 4× field (5 mm in diameter)
No solid growth
LVI (±)
Pattern C
Diffuse destructive invasion
Infiltrative glands that are variable in shape and size, often angulated or interconnected
Confluent growth
Glands or papillary structures with little intervening stroma or mucin lakes with tumor cells within the cervical stroma and filling a 4x filed (5mm)
Solid
Poorly differentiated component (architecturally high grade) with sheets of large malignant cells
Extensive linear destructive
Diffuse laminar EACs ≥5 mm. Tumor cells or individual glands are present in a desmoplastic stroma at the base of the tumor
Band-like lymphocytic infiltrate
Superficial prominent band-like lymphoid infiltrate that obscures the neoplastic aggregates
Micropapillary
Numerous small clusters of tumor cells
LVI (±)

* Adapted from Diaz de Vivar et al., Roma et al., and Alvarado-Cabrero et al. 20–22. Adaptations are themselves works protected by copyright. So in order to publish this adaptation, authorization must be obtained both from the owner of the copyright in the original work and from the owner of copyright in the translation or adaptation.

EACs indicates endocervical adenocarcinomas.

#### Pattern A

**FIG. 1:**
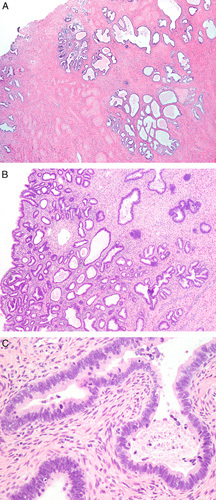
Invasive human papilloma virus–associated adenocarcinoma, pattern A. Proliferation of neoplastic glands focally with lobular architecture. The proliferation extends deep into the cervical stroma (A). The glands have rounded contours (B). Nuclei are elongated and pseudostratified with apical mitoses and lack high-grade cytologic atypia (C).

**FIG. 2:**
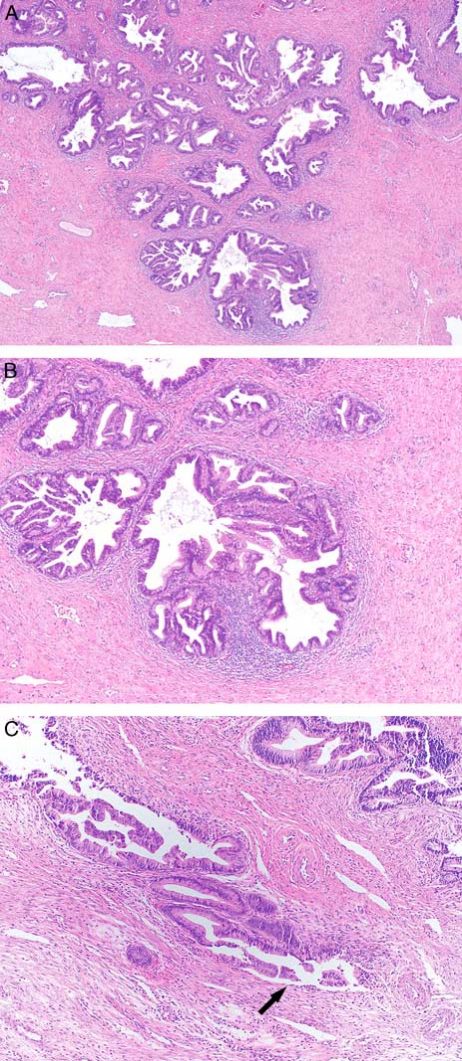
Invasive human papilloma virus–associated adenocarcinoma, pattern A. Diffuse proliferation of neoplastic glands with rounded contours, focal intraglandular papillary architecture, and focal peri-glandular inflammation (A). Notice absence of isolated tumor cells, tumor cell clusters or ragged glands at the base of the glands (B). Glands showing artefactual disruption (see arrow) should not be misconstrued as pattern B (C).

This pattern is characterized by the absence of destructive stromal invasion (i.e. there is no desmoplasia or associated inflammatory infiltrate with single cells or detached clusters of tumor cells within the stroma). It consists of well-demarcated glands with rounded contours, commonly forming groups that sometimes have a relatively well-preserved lobular architecture (Figs. 1, 2). This pattern should not be diagnosed in the presence of high-grade cytologic features or solid architecture. Although cribriform or papillary growth may be seen, these should not fill a 4× field (5 mm in diameter). As stated above, this cut-off is not evidence based and was obtained by consensus of the investigators who developed this system. LVI is absent in pattern A, thus when considering assigning this pattern, close scrutiny should be carried out in order to exclude this finding, this may require levels or immunohistochemical studies. As destructive invasion needs to be excluded, a pattern A designation requires examination of the entire tumor on excisional material [eg, loop electrosurgical excision procedure (LEEP) or cone] with negative resection margins.

#### Pattern B

**FIG. 3:**
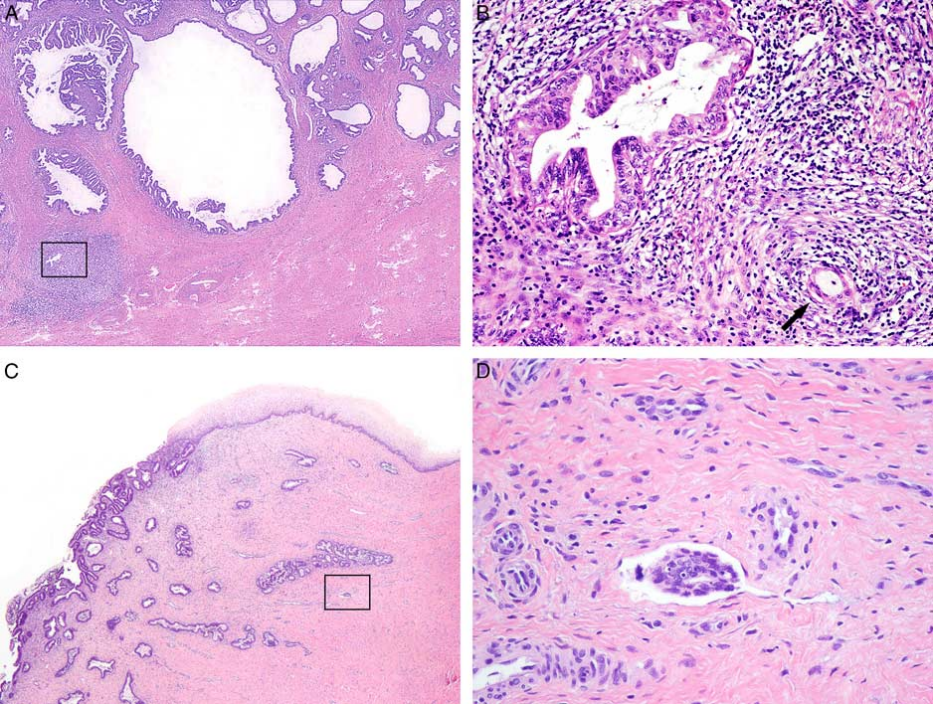
Invasive human papilloma virus–associated adenocarcinoma, pattern B. Diffuse proliferation of neoplastic glands with a rounded contour and focal intraglandular papillary architecture, and small gland with focal inflammation, square (A), higher magnification shows a small gland with flattened epithelium indicating early destructive invasion, arrow (B). Mostly diffuse proliferation of neoplastic glands extending into the ectocervix (C) and with focal lymphovascular invasion, square (D).

**FIG. 4:**
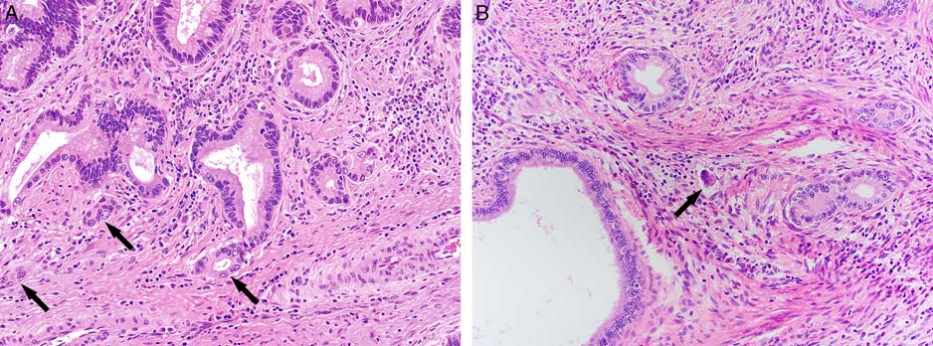
Invasive human papilloma virus–associated adenocarcinoma, pattern B. Ragged glands and tumor cells clusters budding off rounded glands (arrows) (A). Focal lymphovascular invasion can be seen (arrow) (B).

This pattern’s hallmark is the presence of localized (early, limited) destructive stromal invasion. It consists of tumor nests, ragged glands or individual cells budding off well-demarcated glands (pattern A) and usually associated with an inflamed or desmoplastic stroma. Foci of localized destructive invasion may be single, multiple, or linear at the base of tumor, but they should not fill a 4× field (5 mm in diameter). No solid growth is seen while LVI may be present or absent (Figs. 3, 4).

#### Pattern C

**FIG. 5:**
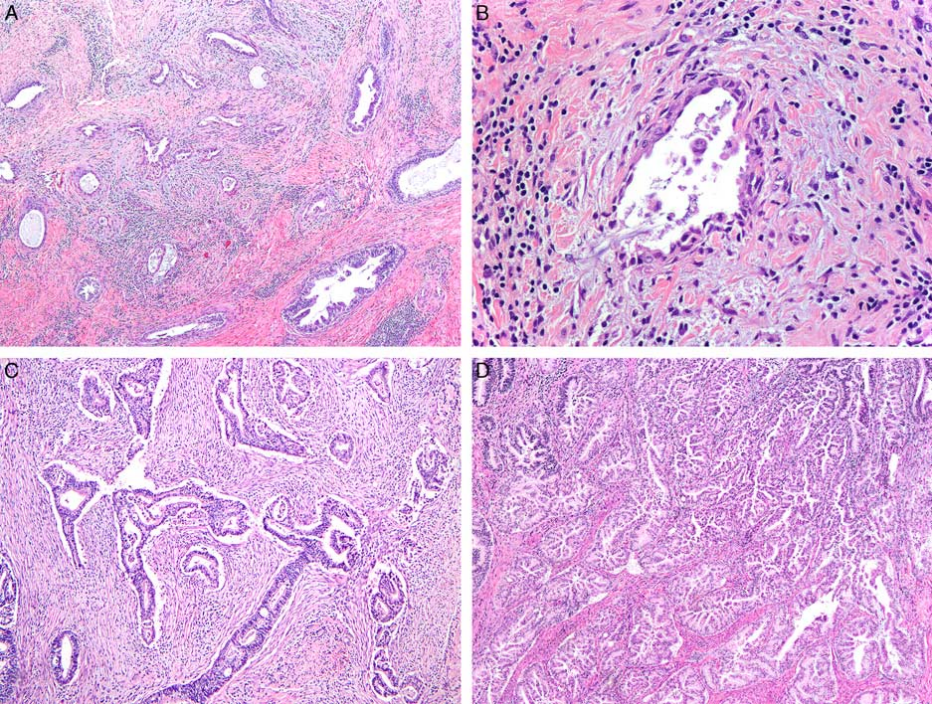
Invasive human papilloma virus–associated adenocarcinoma, pattern C. Diffuse proliferation of variably sized and shaped glands, many of them angulated, in a desmoplastic stroma (A), some of the glands are elongated and with flattened epithelium, microcystic, elongated and fragmented–like (B). Interconnected glands in a desmoplastic stroma (C), confluent glands with intraglandular papillary growth filling a 4× field (5 mm) (D).

The presence of diffuse destructive stromal invasion is the cardinal feature of this pattern. Tumors with pattern C can show any of the following histological appearances:

1. A growth of haphazardly distributed, variably sized and shaped, often angulated glands in a desmoplastic stroma; the glands can be interconnected (canalicular pattern), and sometimes they are interspersed with dilated, elongated, and fragmented glands that resemble those seen in the microcystic, elongated and fragmented pattern of invasion of endometrial endometrioid carcinomas (Figs. 5A–C).
2. A confluent glandular or papillary growth with minimal intervening stroma—endophytic growth only, or mucin lakes with tumor cells filling a 4× field (5 mm in diameter) (Fig. 5D).
3. A micropapillary growth with small papillae composed only of tumor cells, lacking fibrovascular cores, and surrounded by clear spaces.
4. A linear proliferation of irregular glands and individual cells in a desmoplastic stroma at the base of a partially exophytic tumor and filling a 4× field (5 mm in diameter).
5. A proliferation of irregular glands or tumor cell aggregates surrounded by an extensive and dense band-like inflammatory infiltrate at the base of a tumor and filling a 4× field (5 mm in diameter).
6. A solid growth of tumor cells with small or abortive glands.

LVI may be present or absent and it is not crucial for the diagnosis of pattern C.

Interestingly, the micropapillary variant of pattern C has been found to be associated with large tumors, and a high propensity to have lymph node metastasis, recurrences, and an adverse outcome 22–25. In addition, some of the investigators involved with the development of the Silva classification have recently published data that appear to indicate differences in the biologic behavior of tumors within the pattern C category; for example, tumors with a diffuse, destructive growth pattern have a tendency to recur while tumors with a band-like lymphocytic infiltrate or extensive linear destructive invasion do not. Also, patients with tumors showing a mixed diffuse and confluent destructive invasion had a worse 6-yr overall survival than patients with other subtypes of pattern C tumors 21. However, additional studies are needed to confirm these findings.

**FIG. 6:**
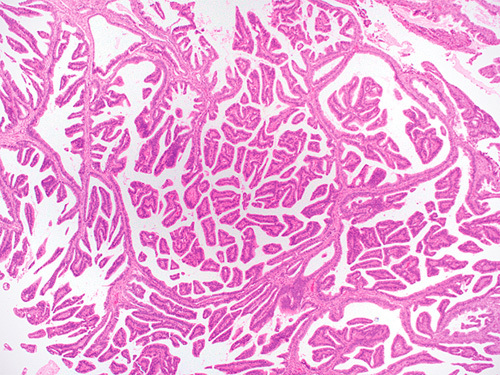
Invasive human papilloma virus–associated adenocarcinoma, pattern C. Confluent villoglandular growth within the cervical stroma filling a 4× field (5 mm).

Pathologists using the Silva classification must be aware of the following:

1. The worst pattern seen in a given tumor is the one to be reported (i.e. tumors with pattern B and focal pattern C, should be classified as pattern C).
2. In exophytic tumors the Silva pattern is evaluated at the tumor base within the cervical wall and not within the exophytic portion of the neoplasm. For example, an exophytic tumor with a villoglandular pattern should not be classified as a pattern C, even if complex, if the invasion at the interface with the underlying cervical wall is nondestructive (therefore, a pattern A tumor). In contrast, if the invasion at the interface with the underlying stroma shows a confluent pattern filling a 4× field (5 mm), the tumor is classified as a pattern C tumor (Fig. 6). It is worth noting that exophytic lesions are challenging because their gross size by itself might determine the stage according to the current FIGO system 11,12.

### Silva Pattern-based Classification: Clinical Impact

We performed an exhaustive literature search using PubMed (US Library of Medicine, Bethesda, MD) and EMBASE. The search included studies published on or before February 2020, using the keywords “adenocarcinoma,” “cervix” or “endocervical,” and “pattern.” Since the first description of the Silva classification, 9 studies reporting on the subgrouping of HPV-associated EACs by pattern of invasion and outcome have been published in the English literature 21,26–33. These 9 studies amount to a total of 1319 patients with invasive endocervical adenocarcinoma. A summary of their distribution by pattern is shown in Table 3 while rates of lymph node metastases, stage distribution, recurrence, and cancer-related deaths are summarized in Table 4.

**TABLE 3:** Studies published from 2013 to 2020 on endocervical adenocarcinoma categorized as per the Silva pattern-based classification

References	No. Pts	Pattern A	Pattern B	Pattern C
Diaz De Vivar et al. 20	352	73 (20.7%)	90 (25.5%)	189 (56.6%)
Djordjevic et al. 26	47	10 (21.3%)	17 (36.2%)	20 (42.5%)
Hodgson et al. 27	20	5 (25%)	6 (30%)	9 (45%)
Stolnicu et al. 33	292	43 (14.7%)	27 (9.24%)	222 (76.02%)
Spaans et al. 28	82	18 (22%)	30 (37%)	34 (41%)
Wang et al. 29	191	46 (24.08%)	41 (21.5%)	104 (54.5%)
Byun et al. 30	63	20 (31.7%)	18 (28.5%)	25 (39.6%)
Xu et al. 31	201	28 (13.9%)	21 (10.4%)	152 (75.6%)
Rivera-Colon et al. 32	71	10 (14%)	12 (16.9%)	49 (69%)
Total	1319	253 (19%)	262 (20%)	804 (61%)

**TABLE 4:** Cumulative clinico-pathologic features of patients with endocervical adenocarcinoma categorized according to the Silva pattern-based classification

	Total	Pattern A	Pattern B	Pattern C
No. Pts	1319	253	262	804
LVI	543 (41%)	0	53 (20%)	490 (61%)
With LN mets	191 (14%)	0	14 (5%)	177 (22%)
With stage information	1102	224	241	637
Stage I	981 (89%)	222 (99%)	233 (97%)	526 (83%)
Stage II–IV	119 (11%)	2 (1%)	6 (3%)	111 (17%)
With F/U	776	201	216	359
F/U in months, mean (range)	62 (3–392)	62 (3–252)	69 (5–392)	55 (3–258)
With recurrences	77 (10%)	0	7 (3%)	70 (19%)
DOD	42 (5%)	0	3 (1%)	39 (11%)

DOD indicates dead of disease; F/U, follow-up; LN, lymph node.

A total of 253 (19%) patients had tumors with pattern A. None had lymph node metastases. Stage information was available in 224 patients of which 222 (99%) had stage I tumors at presentation. A total of 201 patients had available follow-up (range, 3–352 mo; median, 62 mo) and none had documented recurrences or cancer-related deaths.

A total of 262 (20%) patients had tumors with pattern B. Fifty-three (20%) had LVI, and 14 (5%) had lymph node metastases. Of the 239 patients with stage information, 233 (97%) had FIGO stage I, and 6 (2.5%) stage II tumors. Among 216 patients with follow-up (range, 5–392 mo; median, 69 mo) 7 (3%) developed recurrences: 2 patients exhibited locoregional recurrences, while 1 each developed ovarian and vaginal recurrence; information was not available in the remaining 3 patients. Three (1%) patients died of disease.

A total of 804 (61%) patients had pattern C tumors. LVI was present in 490 (61%) tumors, and lymph node metastases in 177 (22%). Compared with patients with pattern A and pattern B adenocarcinomas, the proportion of patients with stage I disease in this group was lower (526 of 789 cases with staging information available, 65%). Among the 359 patients with follow-up (range, 3–258 mo; median, 55 mo), 70 developed recurrences (19%), 11 in the vagina, 6 were locoregional, and approximately half had distant metastases. Cancer-related death occurred in 39 (11%) patients.

Current evidence, while retrospective, supports the use of the Silva classification for the clinical management of patients with HPV-associated invasive adenocarcinomas. The differences in outcome suggest that patients with pattern A adenocarcinomas can be treated conservatively with conization with negative margins and no lymph node dissection, similar to patients with adenocarcinoma in situ (AIS). Follow-up of these patients is still required as rare examples of cervical tumors with an in situ adenocarcinoma appearance have been associated with ovarian metastasis 34. In contrast, patients with pattern B tumors with LVI may benefit from SLN mapping or a limited LN sampling. This recommendation differs from an initial recommendation where all patients with pattern B tumors were thought to benefit from SLN mapping. Currently, it is felt that patients with pattern B tumors with no LVI should be treated as those with pattern A tumors. Patients with pattern C tumors have the highest prevalence of adverse outcomes, and therefore are more likely to benefit from standard surgical treatment including SLN sampling or LN dissection. Substratification of pattern C into variants with less (extensive linear, band-like lymphocytic) versus more aggressive (diffusely destructive or confluent, micropapillary) behavior may help in the future to choose specific management strategies 22–25. The role of systemic therapy (chemotherapy and/or radiation) in patients stratified by pattern of invasion is, to date, unclear.

**Table:** 

Summary of evidence: pattern-based classification and patient outcome	Level of evidence
Pattern A adenocarcinomas have no risk of lymph node metastases, and a negligible risk of recurrence and cancer-related mortality* Pattern B adenocarcinomas have a low (<4%) risk of nodal metastases, recurrence and cancer-related mortality Pattern C adenocarcinomas have a ~20% risk of nodal metastases and tumor recurrence, as well as a ~10% risk of cancer-related mortality	2A

*Long-term follow-up of these patients is still required.

### Reproducibility

The interobserver reproducibility of the Silva pattern-based classification has been addressed by 3 independent studies to date. The first study included 2 institutions and 49 cases of usual type invasive adenocarcinoma 4. The investigators found consensus diagnosis in 50% of cases, with kappa values ranging from fair to almost perfect agreement (range, 0.24–0.84); kappa agreement improved when using a 2-tier system (pattern A vs. pattern B or C). The second study was multi-institutional, included 96 cases and found a good overall reproducibility (κ=0.65). While perfect agreement (9/9 reviewers) was seen in only 11 cases (11%), consensus (≥5/9 reviewer) concordance was achieved in 82/96 cases (85%). Interobserver agreement was the highest when distinguishing in situ adenocarcinoma and pattern A from pattern B and C tumors. Poor agreement was seen in the distinction between in situ adenocarcinoma and pattern A adenocarcinoma 35. The third study was also muti-institutional, encompassed 84 cases, and found an overall concordance of 74% with kappa values of 0.54, 0.32 and 0.59 for patterns A, B, and C, respectively 36.

We conclude that the Silva pattern-based classification has overall an acceptable reproducibility, especially when distinguishing pattern A from pattern B or C tumors. Pathologists are encouraged to become proficient in using this classification by completing the ISGyP training module on the Silva classification (http://www.gpecimage.ubc.ca/aperio/images/eac/). This resource offers training and test sets of cervical adenocarcinomas classified by pattern of invasion. Lastly, routine intradepartmental consultation and consensus opinion with colleagues, at least in difficult cases, can be helpful.

**Table:** 

Summary of evidence: interobserver reproducibility of the pattern-based classification	Level of evidence
Reproducibility of the pattern-based classification is acceptable	Not applicable
Agreement increases when distinction is between pattern A (nondestructive) and patterns B or C (destructive)	Not applicable

### Current Issues and Recommendations

#### Reporting of Pattern of Invasion

The Silva classification is not part of the current FIGO or American Joint Commission on Cancer (AJCC) staging systems 11. Nonetheless, it is now mentioned in synoptic reporting guidelines such as the College of American Pathologists (as a fillable field under “Stromal Invasion”) and the International Collaboration on Cancer Reporting (as an explanatory note under “grading”) 37,38. Moreover, the latest National Comprehensive Cancer Network (NCCN) guidelines introduce the Silva classification as an “emerging concept” 15.

We recommend including these patterns of invasion in the pathology reports with a diagnosis of invasive HPV-associated endocervical adenocarcinoma. The pattern of invasion can be included as a subheading of the main diagnosis line, or in the comment section. The former is preferred by this group. Including an explanatory note can also be considered.

**Table:** 

Recommendations: pattern-based classification reporting	Grade of recommendation
The pattern-based classification should be applied to all invasive HPV-associated endocervical adenocarcinomas	B
The pattern should be included in the Final Diagnosis and/or Comment sections of the surgical pathology report	
If pattern C is identified, the presence of the micropapillary subtype should be reported	C
To increase reproducibility, completion of training modules (such as the ISGyP online module on pattern-based classification) and intradepartmental/interdepartmental consultation with colleagues is encouraged	D

#### Specimen Type and Silva Pattern-based Classification

A prerequisite for the application of the Silva classification is the histologic examination of the entire tumor. Thus, pattern assignment is best done in a cone or LEEP with negative margins, or in a hysterectomy or trachelectomy specimen. Biopsy material is not suitable for pattern assignment given its limited size and superficial nature 26,32. Conversely, it has been shown that the Silva pattern of invasion in LEEP and cone material is highly predictive of the overall pattern of residual tumor in hysterectomy) 26,32.

**Table:** 

Summary of evidence: Silva pattern-based classification and specimen type	Level of evidence
Prediction of the overall pattern based on biopsy material alone is suboptimal with significant chance of change on final excision	4
Classification is applicable to excisional material (loop electrosurgical excision procedure, cone, trachelectomy, hysterectomy)	4

Recommendations: pattern-based/Silva classification and specimen type	Grade of recommendation
On excisional specimens, application of the Silva system requires exhaustive microscopic examination of the tumor	C
On excisional material, a pattern A designation requires first examination of the entire tumor (to exclude destructive invasion)	C
In biopsy material: If present, pattern C can be reported Pattern A or B designation is not recommended	C

#### LVI and the Silva Pattern-based Classification

LVI is an important parameter in the management of cervical cancer. However, not all studies support its independent prognostic significance, especially in multivariate analyses. Creasman and Kohler 39 reviewed the published literature encompassing 25 studies with data on 6500 patients with early cervical cancer and LVI; only 3 (12%) studies found LVI as an independent risk factor. In a study focused on 127 patients with pattern C EACs. Roma et al. 40 found that LVI was not an independent predictor of survival. Despite this evidence, it is still important to report the LVI status as it currently affects patient management. In terms of the value of quantifying LVI in cervical adenocarcinoma, a study of 189 pattern C tumors showed that the extent of lymphatic vascular invasion may have prognostic significance, as those with extensive LVI (≥20 individual spaces containing tumor) had significantly higher rates of lymph node metastases and recurrence compared to those with low volume LVI (0–4 spaces) 22. This evidence suggests a potential role for quantifying the extent of LVI similar to endometrial carcinoma. However, further studies are needed to confirm this finding.

**Table:** 

Summary of evidence: lymphovascular space invasion	Level of evidence
Lymphovascular space invasion is not an independent prognostic factor in pattern C adenocarcinoma	2A
Quantification of the amount of lymphovascular invasion may improve the prognostic value of this parameter	2B

Recommendations: lymphovascular space invasion	Grade of recommendation
As it influences management, it is recommended to report the lymphovascular invasion (LVI) status in all patients with pattern B and C endocervical adenocarcinoma	C
A quantitative estimation of LVI extent can be included in a comment (number of foci)	C

#### HPV-independent Adenocarcinoma and the Silva Pattern-based Classification

The Silva classification was conceived using cohorts of usual-type adenocarcinomas, and it is applicable to this tumor type as outlined in the seminal study by Diaz de Vivar et al. 20. It is also applicable to other types of HPV-related adenocarcinoma as recently demonstrated by Stolnicu et al. 33. Conversely, patients with HPV-independent adenocarcinomas, gastric-type being most common, do not benefit from pattern-based stratification as most show pattern C invasion even when well-differentiated (namely gastric-type adenocarcinoma, minimal deviation type) and are associated with poor prognosis.

**Table:** 

Summary of evidence: Silva pattern-based classification in HPV-independent endocervical adenocarcinoma	Level of evidence
HPV-independent adenocarcinomas are aggressive tumors regardless of the growth pattern. Most tumors have a pattern C of invasion	2B

Recommendation: Silva pattern-based classification in HPV-independent endocervical adenocarcinoma	SR
Silva pattern-based classification applies only to HPV-associated invasive endocervical adenocarcinomas Silva pattern-based classification is not recommended in HPV-independent adenocarcinomas	B

## DISTINCTION BETWEEN ADENOCARCINOMA IN SITU AND INVASIVE ADENOCARCINOMA

### HPV-associated Endocervical Adenocarcinoma

#### Definitions

HPV-associated AIS is defined as a proliferation of neoplastic glandular cells confined to the epithelial endocervical compartment and related to infection by high-risk HPV. From a histopathologic perspective, HPV-associated AIS is defined by the following criteria 41–43.

**FIG. 7:**
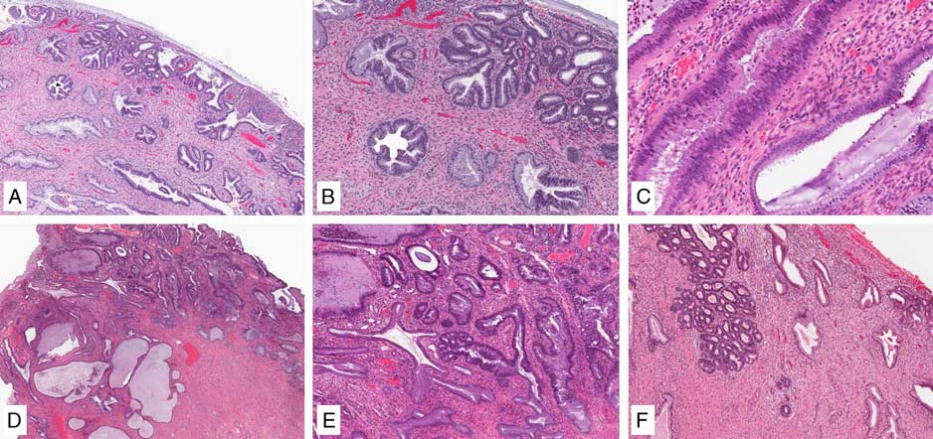
Human papilloma virus–associated adenocarcinoma in-situ is defined as a population of neoplastic glandular epithelium replacing the non-neoplastic endocervical epithelium with preservation of pre-existing glandular architecture (A, B). Partial crypt/glandular involvement can be seen (C). Comparison with the adjacent normal endocervix is often useful in setting a threshold for the diagnosis of adenocarcinoma in situ (and separating from invasive adenocarcinoma), which is important as the normal endocervix can be crowded (D, E). Lobulated growth can be accepted in adenocarcinoma in situ (F).

**FIG. 8:**
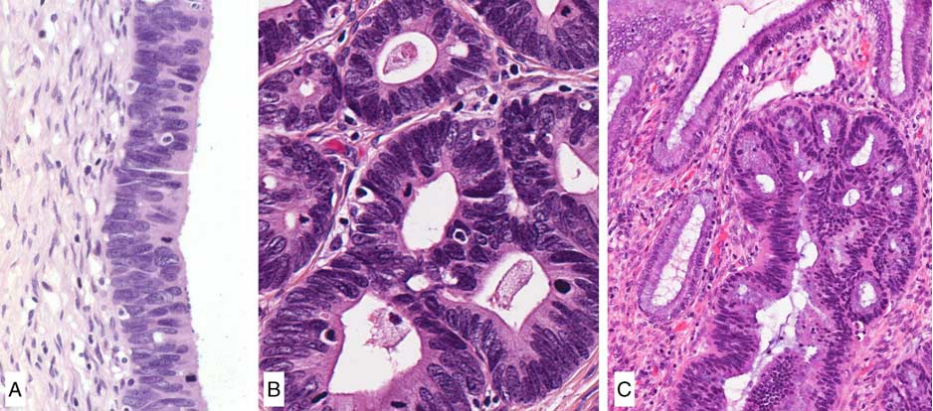
Human papilloma virus–associated adenocarcinoma in-situ is characterized by conspicuous mitoses and apoptosis; in its most common, known as usual-type, neoplastic cells have mucin depletion and enlarged, elongated hyperchromatic nuclei with an increased nuclear-to-cytoplasmic ratio (A, B). Complex cribriform growth is allowed in adenocarcinoma in situ, as long as it is intraglandular only (C).

- Architecture: as the neoplastic cells are replacing the preexisting normal endocervical cells, there is preservation of the normal glandular architecture. Intraglandular and/or surface architectural complexity is allowed (papillary, micropapillary or cribriform growth), but should be limited (Fig. 7).
- Cytology: columnar cells with enlarged, elongated or plump, hyperchromatic nuclei, mucin-depleted (more often) or mucin rich epithelium, and easily identifiable apical mitotic figures and apoptotic bodies (at least one in each gland). Nuclear stratification is common (Fig. 8).

**FIG. 9:**
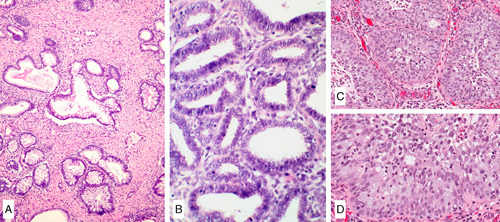
Human papilloma virus–associated adenocarcinoma in situ variants. Intestinal-type adenocarcinoma in situ has mucinous goblet cell differentiation (A). The exceedingly rare tubal-type adenocarcinoma in-situ features apical snouts resembling tubal epithelium (B). Stratified mucin producing intraepithelial lesion (SMILE) is characterized by mucinous cells arranged in multiple layers, narrowing the lumen of the endocervical crypt and mimicking a squamous lesion (C), the atypical nuclei and mucinous cytoplasm of the lesional cells can be appreciated throughout the epithelial thickness (D).

*Histologic Variants*. The most common HPV-related AIS is the usual type, which is similar to its invasive counterpart. Less commonly, HPV-related AIS is of intestinal type featuring goblet cell differentiation. The stratified variant is also known as stratified mucin-producing intraepithelial lesion—SMILE 44. Other variants described in literature before IECC include endometrioid and tubal. The term endometrioid no longer applies to the spectrum of HPV-associated endocervical adenocarcinoma, and its use is discouraged as the vast majority are thought to represent mucin depleted HPV related in situ adenocarcinomas 45–47. Similarly, tubal AIS is poorly characterized in the literature although it may arise from tubal metaplasia within the cervix 47 (Fig. 9).

HPV-associated invasive endocervical adenocarcinoma is defined as a proliferation of neoplastic glandular cells, related to infection by high-risk HPV, and showing cervical stromal invasion. Invasion of the cervical stroma is characterized by 43,48:

- Infiltrative/destructive growth: glands with irregular or angulated contours; desmoplastic stromal reaction; non–gland-forming elements (individual cells, cell clusters, buds or nests).
- Complex confluent growth: anastomosed, fused or interconnected glandular elements with scant to no stroma in between; complex cribriform, labyrinth-like or solid patterns occupying a 4× field (5 mm in diameter).

**FIG. 10:**
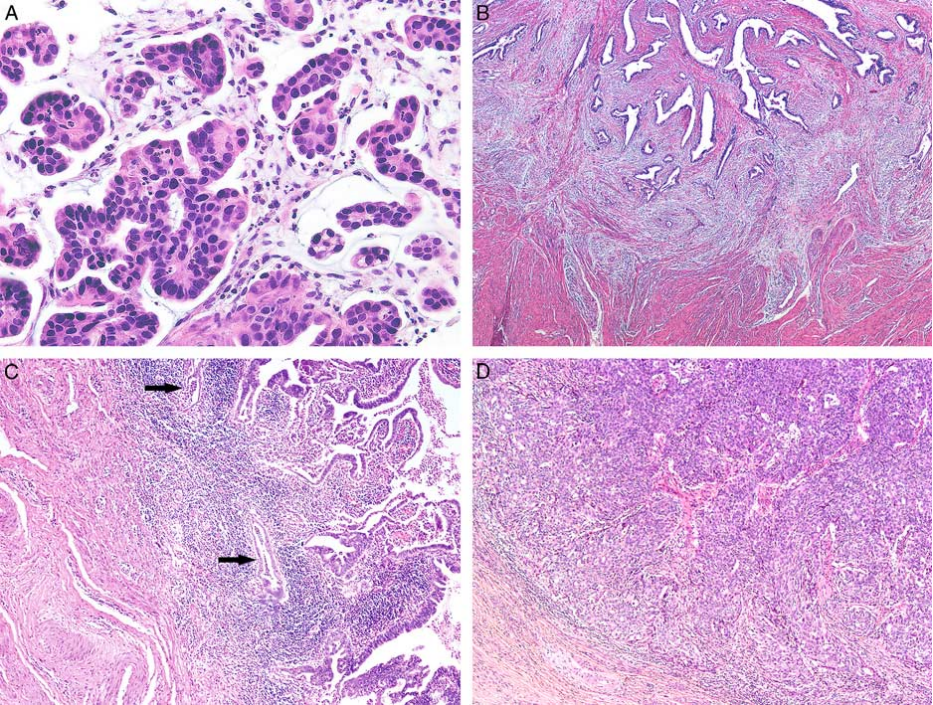
Invasive human papilloma virus–associated adenocarcinoma, pattern C. Micropapillae in spaces devoid of lining (A). Linear proliferation of irregular glands in a desmoplastic stroma at the tumor base and filling a 4× field (5 mm) (B). Elongated glands, arrows, and dense lymphocytic infiltrate filling a 4× field (5 mm) (C). Solid pattern with small glands (D).

Under the Silva classification, the features described above define “destructive” types of invasion, namely patterns B and C (see The Silva pattern-based classification: definitions section) (Figs. 3–6, 10).

A “nondestructive” or “AIS-like” pattern of growth has also been historically classified as a form of invasive carcinoma. This type of invasion is characterized by:

- Increased glandular density: gland crowding that deviates from the normal endocervical crypt distribution; tight clustering of small glands, sometimes with a lobulated appearance, and lacking high-grade nuclear features.
- Deep glandular proliferation: glands with a haphazard distribution present in deep cervical stroma without stromal reaction often in close proximity to thick-walled vessels.

This growth is analogous to pattern A (Figs. 1, 2). The cytologic features of HPV-related invasive adenocarcinoma are the same as described previously for HPV-related AIS 44.

#### Current Issues

*Distinction Between In Situ and Invasive Adenocarcinoma*. The reproducibility in distinguishing in situ and invasive endocervical adenocarcinoma is fair to poor 35. In fact, it has been estimated that such distinction cannot be made in as much as 20% of cases 49. The lowest degree of interobserver agreement is observed between AIS and pattern A adenocarcinoma 35. The architectural overlap between AIS and nondestructive invasive adenocarcinomas that lacks stromal reaction may explain the inconsistent interobserver agreement 50, although evaluating the pre-existing adjacent endocervical glands may be helpful in deciding how much complexity can be allowed to establish a diagnosis of AIS.

To this point, it is important to remember that the endocervical mucosa as a complex system of mucosal infoldings, first described by Fluhmann 51,52. The basic structural unit of the endocervix is an array of haphazardly distributed epithelial infoldings (clefts and grooves) rather than a vertical tubular gland as occurs in the endometrium. The haphazard orientation of these infoldings results in a heterogeneous, or “pattern-less,” appearance which contributes to our established limitation in distinguishing in situ adenocarcinoma (occupying pre-existing endocervix) from invasive adenocarcinomas.

The biologic behavior of nondestructive (pattern A) adenocarcinoma is indolent. As discussed previously, of a total of 253 patients with pattern A adenocarcinoma reported in the literature to date, none have associated lymph node metastases 21,26–33. Moreover, no recurrences or cancer-related deaths were documented among the 201 patients reported with available follow-up (mean follow-up period 62 mo, range, 3–262 mo) 21,26–30. Given the excellent outcome of patients with tumors showing pattern A, mirroring the behavior as AIS, it has been suggested to lump pattern A tumors as part of the AIS category. However, ovarian spread has been documented in adenocarcinomas with reported AIS-like growth pattern 34,53. In a series of 29 patients with endocervical adenocarcinoma and synchronous or metachronous ovarian metastases reported by Ronnett et al. 34, 11 had AIS-like appearance. The study included tumors with superficial or subtle invasion, comprised of “haphazardly distributed smaller glands in a pattern more extensive than typical AIS,” or foci suspicious but not unequivocal for invasion. Of note, a subset of cases in this study underwent review of only representative slides, not the entire histologic material. While there are no reports of pattern A tumors with ovarian metastases in the Silva classification literature, ovarian status has not usually been specified, thus a definitive statement about pattern A tumors and their potential risk of ovarian metastasis cannot be provided. It can be inferred that tumors with ovarian spread and AIS-like growth reported represent pattern A lesions; however, this needs to be confirmed by further studies that describe the true prevalence of ovarian metastases in lesions defined as per the Silva system. Therefore, it is advisable to follow-up patients with pattern A cervical adenocarcinomas.

**Table:** 

Summary of evidence: distinction between in situ and invasive HPV-associated endocervical adenocarcinoma	Level of evidence
Distinction between in situ and invasive endocervical adenocarcinoma suffers from fair to poor interobserver agreement Agreement is low in distinguishing between AIS and pattern A adenocarcinoma	2B
Pattern A adenocarcinomas have a nil risk of nodal metastases and adverse outcome, mirroring behavior of AIS	2A
Ovarian spread has been reported in tumors with AIS-like growth; further studies are required to determine the prevalence of ovarian metastases in patients with pattern A adenocarcinoma	4

#### Recommendations

**FIG. 11:**
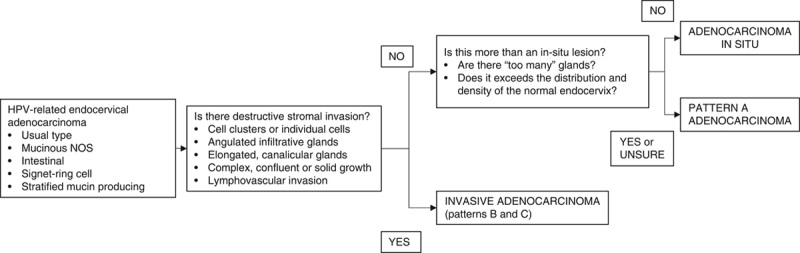
Algorithmic approach to the distinction between in-situ and invasive HPV-associated endocervical adenocarcinoma. HPV indicates human papilloma virus; NOS, not otherwise specified.

On the basis of the above cumulative evidence, and while more data on the risk of ovarian spread by pattern A adenocarcinomas becomes available, we advise against categorizing pattern A adenocarcinomas as AIS. Instead, we recommend an approach that emphasizes the tumor growth pattern, as follows (Fig. 11):

1. Look for *destructive* stromal invasion. If present, diagnosis of “invasive endocervical adenocarcinoma” is appropriate.
2. If destructive invasion is absent, determine if the lesion is within the volume and distribution expected for an in situ lesion; if so, the diagnosis of “AIS” is appropriate.
3. If the lesion exceeds the volume and distribution expected for AIS, or the distinction between AIS and invasive is difficult, the diagnosis of “*pattern-A (non-destructive) adenocarcinoma*” is appropriate.

In the context of marked inflammation, mucosal erosion or ulceration and previous biopsy site reaction, the architecture of the lesion may be distorted. Evaluation of growth and stromal invasion should be made in other areas.

In all the above considerations, consensus review with colleagues and outside consultation are helpful steps to reach a final diagnosis.

**Table:** 

Recommendations:	Grade of recommendation
Distinction between in situ and invasive HPV-associated endocervical adenocarcinoma	
*Invasive adenocarcinoma*: in the presence of destructive growth (patterns B and C), diagnosis of *invasive* adenocarcinoma is warranted	B
In the absence of destructive growth, the following diagnoses should be considered: * Adenocarcinoma in-situ*: In the absence of destructive growth, draw attention to the gland distribution and density; if these are within the confines of the normal endocervix, diagnosis of adenocarcinoma in-situ is warranted * *Comparison with the uninvolved/normal endocervical gland architecture is helpful. * Pattern-A (nondestructive) adenocarcinoma*: if a nondestructive lesion exceeds the size and distribution expected for AIS, or such determination cannot be made, the diagnosis of pattern-A adenocarcinoma (with nondestructive growth) is warranted * *It is recommended for now to separate these lesions from frankly invasive adenocarcinoma, as their behavior is largely indolent * *It is currently not recommended to classify them as AIS until new evidence on their risk of ovarian spread is available. * *Reporting size, stage and margin status is still warranted in this category	C

### HPV-independent Endocervical Adenocarcinoma

According to the new classification of endocervical adenocarcinoma, the HPV-independent category includes gastric, clear cell, mesonephric, and endometrioid types. Among these subtypes, there is emerging evidence on the spectrum of in situ gastric type endocervical neoplasia. In situ counterparts for clear cell, mesonephric and endometrioid carcinomas of the cervix have not been described in the literature.

#### Definitions

**FIG. 12:**
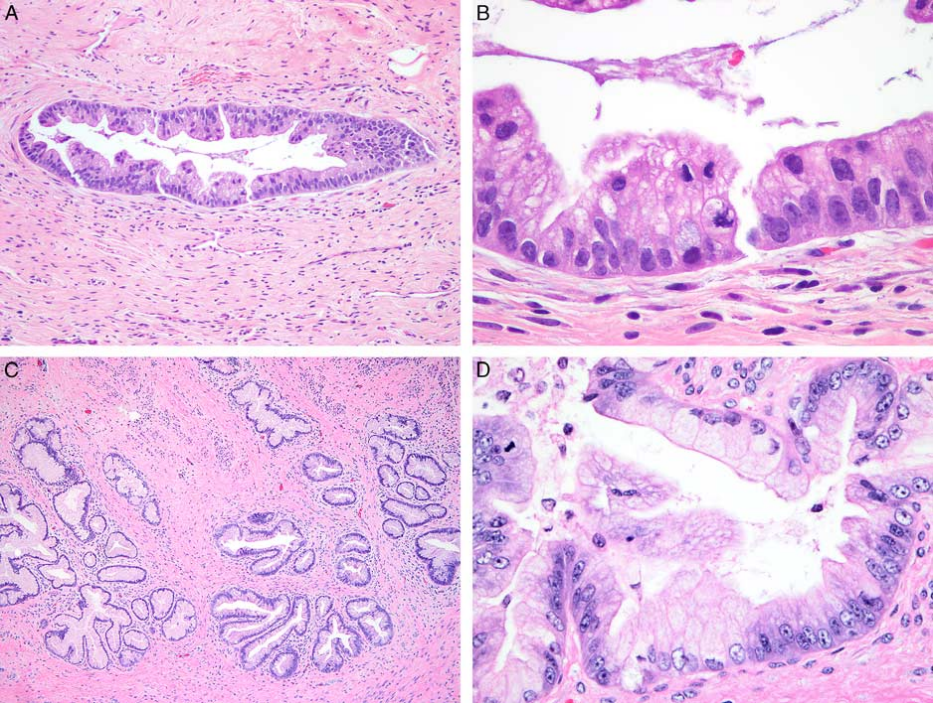
Gastric-type adenocarcinoma in situ, well-demarcated gland and cells showing abundant clear or light eosinophilic cytoplasm, well-defined cell borders, marked nuclear atypia and mitotic activity (A, B). Atypical lobular endocervical gland hyperplasia, with preservation of the lobular architecture (C), and cytologic atypia (D).

*Gastric-type AIS*. This lesion is defined by architectural criteria identical to HPV-related AIS. From a cytomorphology perspective, gastric type AIS is composed of mucinous cells with abundant foamy vacuolated cytoplasm, distinct cell borders and nuclear atypia 54,55 (Figs. 12A, B). Intraglandular complexity, in the form of cribriform, papillary or micropapillary growth, can be seen.

*Atypical Lobular Endocervical Glandular Hyperplasia (LEGH)*. LEGH is a benign glandular proliferation composed of cells with a gastric mucinous phenotype. As the name implies, it has an acinar (lobular) configuration, comprised of a central gland/duct surrounded by smaller round glands arranged in a floret-like pattern 56,57. In contrast to the conventional LEGH, atypical LEGH shows a spectrum of cytologic features 54,58. It has been proposed that this diagnosis requires the presence of at least 4 of the following, in a lesion architecturally consistent with LEGH: nuclear enlargement; irregular nuclear contours; distinct nucleoli and coarse chromatin; loss of polarity; mitoses; apoptotic bodies or luminal nuclear debris; intraluminal papillary projections.

The distinction between A-LEGH and the more recently characterized gastric-type AIS is expected to be subjective, as there is overlap in the established definitions. In order to harmonize nomenclature, we recommend the term gastric-type AIS if the lesion displays significant nuclear atypia or proliferation regardless of the preexisting architecture (Figs. 12C, D).

#### Current Issues

*Distinction Between In Situ and Invasive Gastric Type Adenocarcinoma*. HPV-related adenocarcinoma can display nondestructive and destructive growth patterns, as discussed above. In contrast, the vast majority of HPV-independent adenocarcinomas, including gastric type, have a pattern C of invasion 33,59. Thus, the distinction between in situ and invasive is typically not problematic. However, an important exception is the well-differentiated end of the spectrum of invasive gastric type adenocarcinoma, namely minimal deviation adenocarcinoma. This variant is remarkable for the highly differentiated glands, minimal to absent cytologic atypia, and absence of desmoplastic reaction 60,61. Unlike AIS, LEGH and atypical LEGH, minimal deviation adenocarcinoma features a haphazard distribution of glands which vary greatly in size and shape, lack lobular organization and typically extend to the outer half of the cervical wall. It is important to note that the reproducibility of these criteria has not been thoroughly assessed.

**Table:** 

Summary of evidence: distinction between in situ and invasive HPV-independent endocervical adenocarcinoma	Level of evidence
Gastric-type adenocarcinoma in situ and atypical lobular endocervical glandular hyperplasia are lesions within the spectrum of gastric-type neoplasia, harboring a relationship with invasive gastric-type adenocarcinoma	4
Gastric-type adenocarcinoma displays infiltrative/destructive pattern invasion or a deceptively bland infiltration pattern (known as “minimal deviation adenocarcinoma”)	4

#### Recommendations

In the distinction between in situ and invasive gastric-type adenocarcinoma, we provide the following recommendations:

- If the glandular proliferation is well-differentiated (eg, composed of well-formed glands with smooth round outlines), consider the following scenarios:
  Gastric type AIS: limited to the surface, similar in density and distribution to the normal endocervical glands, overt nuclear atypia.
  Atypical LEGH: floret-like arrangement with small, round glands surrounding a larger, duct-like structure, typically with a superficial location; nuclear atypia is variable but often present.
  Invasive gastric type adenocarcinoma, minimal deviation type: haphazard gland distribution with variation of gland size and shape, as well as lack of lobular architecture; extension into the deep cervical stroma.

**Table:** 

Recommendations:	Grade of recommendation
Distinction between in situ and invasive gastric type endocervical adenocarcinoma	
In the absence of destructive growth, the following diagnoses should be considered: * Adenocarcinoma in situ*: gland distribution and density similar and within the confines of the normal endocervix. Comparison with the uninvolved/normal endocervical mucosa is helpful * Atypical lobular endocervical glandular hyperplasia:* floret-like arrangements with small, acini-like glands surrounding a central duct and nuclear atypia * Invasive gastric type adenocarcinoma, minimal deviation type:* haphazard distribution of glands with involvement of the deep cervical stroma, lack of lobular organization, minimal to absent nuclear atypia	C

## CONCLUSION

It is our hope that these ISGyP-developed recommendations will facilitate the use of the Silva classification for HPV-associated cervical adenocarcinoma and the proper diagnosis of AIS, not only in the setting of patient care, but also in research activities which ultimately will change the staging and management of this disease. It is important to underscore the fact that enough evidence has been accumulated regarding the Silva classification to proceed with prospective studies in close collaboration with our gynecology oncology colleagues to further support the current evidence.
